# Induction of Cell-Mediated Immune Responses in Mice by DNA Vaccines That Express Hepatitis C Virus NS3 Mutants Lacking Serine Protease and NTPase/RNA Helicase Activities

**DOI:** 10.1371/journal.pone.0098877

**Published:** 2014-06-05

**Authors:** Suratno Lulut Ratnoglik, Da-Peng Jiang, Chie Aoki, Pratiwi Sudarmono, Ikuo Shoji, Lin Deng, Hak Hotta

**Affiliations:** 1 Division of Microbiology, Kobe University Graduate School of Medicine, Kobe, Japan; 2 JST/JICA SATREPS Laboratory of Kobe University, Faculty of Medicine, University of Indonesia, Jakarta, Indonesia; 3 Faculty of Medicine, University of Indonesia, Jakarta, Indonesia; University of Sydney, Australia

## Abstract

Effective therapeutic vaccines against virus infection must induce sufficient levels of cell-mediated immune responses against the target viral epitopes and also must avoid concomitant risk factors, such as potential carcinogenic properties. The nonstructural protein 3 (NS3) of hepatitis C virus (HCV) carries a variety of CD4^+^ and CD8^+^ T cell epitopes, and induces strong HCV-specific T cell responses, which are correlated with viral clearance and resolution of acute HCV infection. On the other hand, NS3 possesses serine protease and nucleoside triphosphatase (NTPase)/RNA helicase activities, which not only play important roles in viral life cycle but also concomitantly interfere with host defense mechanisms by deregulating normal cellular functions. In this study, we constructed a series of DNA vaccines that express NS3 of HCV. To avoid the potential harm of NS3, we introduced mutations to the catalytic triad of the serine protease (H57A, D81A and S139A) and the NTPase/RNA helicase domain (K210N, F444A, R461Q and W501A) to eliminate the enzymatic activities. Immunization of BALB/c mice with each of the DNA vaccine candidates (pNS3[S139A/K210N], pNS3[S139A/F444A], pNS3[S139A/R461Q] and pNS3[S139A/W501A]) that expresses an NS3 mutant lacking both serine protease and NTPase/helicase activities induced T cell immune responses to the degree comparable to that induced by the wild type NS3 and the NS3/4A complex, as demonstrated by interferon-γ production and cytotoxic T lymphocytes activities against NS3. The present study has demonstrated that plasmids expressing NS3 mutants, NS3(S139A/K210N), NS3(S139A/F444A), NS3(S139A/R461Q) and NS3(S139A/W501A), which lack both serine protease and NTPase/RNA helicase activities, would be good candidates for safe and efficient therapeutic DNA vaccines against HCV infection.

## Introduction

Hepatitis C virus (HCV) is an enveloped RNA virus that belongs to the genus *Hepacivirus* of the family *Flaviviridae*. The viral genome encodes a single polyprotein of about 3,000 amino acids, which is cleaved by host and viral proteases to generate at least 10 viral proteins, i.e., envelope 1 (E1) and E2, p7, nonstructural protein 2 (NS2), NS3, NS4A, NS4B, NS5A and NS5B. NS3 is a multi-functional protein with a serine protease domain located in the N-terminal one-third and a nucleoside triphosphatase (NTPase)/RNA helicase domain located in the C terminal two-thirds, which are involved in the proteolytic processing of the viral polyprotein and viral RNA replication, respectively [1], [2], [3].

HCV is a major cause of chronic liver disease, such as chronic hepatitis, liver cirrhosis and hepatocellular carcinoma. It is estimated that 180 million people are currently infected with HCV worldwide, and that ca. 70% of them become chronically infected [4], [5]. The recent approval of NS3 serine protease inhibitors for treatment of HCV genotype 1 infection was a great progress in HCV antiviral development, and combination of a protease inhibitor with interferon (IFN) and ribavirin has increased sustained virological response (SVR) in patients [6]. On the contrary, great success has not been achieved in HCV vaccine development; no effective HCV vaccine is available so far, either for a prophylactic or a therapeutic purpose.

While prophylactic HCV vaccines must have capacity to induce protective levels of neutralizing antibodies directed principally to the viral protein E2, effective therapeutic HCV vaccines must elicit strong cell-mediated immune responses against a wide variety of CD4^+^ and CD8^+^ epitopes of the viral origin. NS3 is known to carry a variety of CD4^+^ and CD8^+^ T cell epitopes to induce strong HCV-specific T cell responses, which are correlated with viral clearance and resolution of acute HCV infection [7], [8], [9], [10], [11]. Also, the HCV core protein is known to carry a variety of CD4^+^ and CD8^+^ epitopes [7], [8], [9], [12], [13], [14]. From the antigenic point of view, therefore, NS3 and the core protein would be attractive candidates to be used for therapeutic vaccines that elicit T cell-mediated immune responses against HCV.

Another important aspect to be assessed carefully in vaccine development is a potential risk(s) of the vaccine-derived peptides/proteins of the viral origin, which might impair or deregulate the normal functions of the host cells. For example, the HCV core protein is known to exhibit oncogenic properties in cell culture systems and transgenic mouse models [15], [16], [17]. The NS3 serine protease cleaves the mitochondrial antiviral signaling protein MAVS (also referred to as IPS-1, VISA and Cardif) to blockade the RIG-I- and TLR3/TRIF-mediated signaling for the induction of IFN-β production [3], [18], [19], [20], [21]. Also, NS3 inactivates T cell protein tyrosine phosphatase and modulates epithelial growth factor (EGF) signaling [22]. Moreover, the NS3 NTPase/RNA helicase, which is principally required for HCV RNA replication [1], [2], may concomitantly deregulate cellular RNA helicase-mediated functions, such as DNA replication, RNA transcription, splicing, RNA transport, ribosome biogenesis, mRNA translation, RNA storage and decay [3], [23], [24], [25]. These observations imply the possible involvement of NS3 in the development of hepatocellular carcinoma. Therefore, a vaccine expressing the functionally active core protein or NS3 may be disadvantageous to the vaccinees. To avoid those potential risks, we introduced a variety of point mutations that abolish the serine protease and NTPase/RNA helicase activities of NS3. We report here that a DNA vaccine that expresses an NS3 mutant lacking both serine protease and NTPase/RNA helicase activities induced strong cell-mediated immune responses in mice, with a high level of IFN-γ production and strong cytotoxic T lymphocyte (CTL) activities.

## Materials and Methods

### Plasmid Construction

Plasmids expressing the entire sequences of wild type NS3 (pSG5-NS3wt) and the NS3/4A complex (pSG5-NS3/4A) of the HCV MKC1a strain (genotype 1b) were derived from the previously reported ones, pcDNA3.1/NS3F(MKC1a) [26] and pcDNA3.1/MKC1a/4A [27], respectively, with the Myc-His tag deleted, and subcloned into the pSG5 vector (Stratagene, USA). To express a polyprotein consisting of full-length NS5A and C-terminally truncated NS5B (NS5A/5BΔC) as a substrate for the NS3 serine protease, the corresponding region of pTM1-NS5A/5BΔC [27] were subcloned into the pSG5 expression vector (Stratagene). Plasmids for production of glutathione S-transferase (GST) and GST-fused NS3 (GST-NS3) were also described previously [26]. An NS3 expression plasmid in the backbone of pEF1/Neomycin(+) (Invitrogen, NY), pEF1/Neo-NS3, was constructed. pIFNβ-Luc, which contains firefly luciferase reporter gene under the control of the interferon β promoter, was a kind gift from Dr. T. Fujita (Kyoto University, Kyoto, Japan) [28]. pRL-TK (Promega), which expresses Renilla luciferase, was used as an internal control. To express an N-terminal part of retinoic acid-inducible gene I (N-RIG-I) [28], the corresponding genomic region was amplified by RT-PCR from Huh-7 cellular RNA and subcloned into an expression vector to generate pEF1A/N-RIG-I-FLAG. pSG5-NS4A was described previously [27].

**Figure 1:**
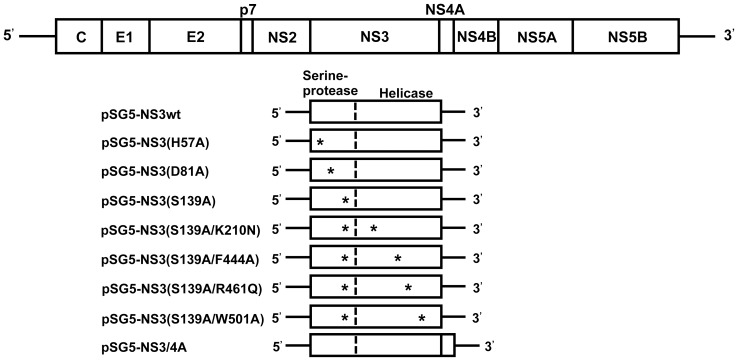
Schematic representation of the HCV genome and the NS3 region with various point mutations. The HCV genome (top) as well as NS3wt and various NS3 mutants are shown. Asterisks indicate point mutations in the serine protease and NTPase/RNA helicase domains.

Single-point mutations were introduced by site-directed mutagenesis into each of the catalytic triad of the NS3 serine protease [29], [30], [31], [32], [33] to generate pNS3(H57A), pNS3(D81A) and pNS3(S139A) that express NS3 mutants lacking the serine protease activity (Fig. 1). Additional mutations, which have been reported to abolish the NTPase/RNA helicase activities of NS3 [34], [35], [36], were introduced into pNS3(S139A) to generate pNS3(S139A/K210N), pNS3(S139A/F444A), pNS3(S139A/R461Q) and pNS3(S139A/W501A). The primers used for the site-directed mutagenesis are shown in Table 1. Introduction of proper mutations were verified by DNA sequencing.

**Table 1:** Primers used for the introduction of HCV NS3 mutations.

NS3 mutation	Position	Sequence*	Direction
H57A	nt 154 to 182	5′-TGTTGGACTGTCTATGCTGGTGCCGGCTC-3′	Forward
		5′-GAGCCGGCACCAGCATAGACAGTCCAACA-3′	Reverse
D81A	nt 229 to 258	5′-AATGTAGACCAAGCCCTCGTTGGCTGGCCG-3′	Forward
		5′-CGGCCAGCCAACGAGGGCTTGGTCTACATT-3′	Reverse
S139A	nt 401 to 430	5′-ACCTGAAGGGTTCCGCGGGTGGTCCGCTGC-3′	Forward
		5′-GCAGCGGACCACCCGCGGAACCCTTCAGGT-3′	Reverse
K210A	nt 616 to 647	5′-ACTGGCAGCGGCAACAGCACCAAGGTGCCGGC-3′	Forward
		5′-GCCGGCACCTTGGTGCTGTTGCCGCTGCCAGT-3′	Reverse
F444A	nt 1315 to 1347	5′-AGCTTGGACCCTACTGCCACCATCGAGACGACG-3′	Forward
		5′-CGTCGTCTCGATGGTGGCAGTAGGGTCCAAGCT-3′	Reverse
R461Q	nt 1369 to 1401	5′-TCGCGCTCGCAGCAGCGAGGCAGGACTGGTAGG-3′	Forward
		5′-CCTACCAGTCCTGCCTCGCTGCTGCGAGCGCGA-3′	Reverse
W501A	nt 1484 to 1517	5′-ATGACGCGGGCTGTGCTGCGTACGAGCTCACGCC-3′	Forward
		5′-GGCGTGAGCTCGTACGCAGCACAGCCCGCGTCAT-3′	Reverse

*The mutated residues in the primer sequences are underlined; nt, nucleotide.

### Cells and Protein Expression

The human hepatoma cell line Huh-7.5 [37] was kindly provided by Dr. Charles M. Rice (The Rockefeller University, New York, NY, USA). Huh-7 and Huh-7.5 cells were cultured in Dulbecco’s modified Eagle’s medium (DMEM) (high glucose) supplemented with 2 mM L-glutamine, 0.1 mM non-essential amino acids (Invitrogen), 50 IU/ml penicillin, 50 µg/ml streptomycin and 10% heat-inactivated fetal calf serum (FCS; Biowest, France) at 37°C in a 5% CO_2_ incubator. For ectopic protein expression, Huh-7.5 cells were transfected with the respective plasmids using X-tremeGENE 9 DNA Transfection Reagent (Roche, Mannheim, Germany) and cultured for 24 to 48 h. Protein expression was confirmed by immunoblotting and indirect immunofluorescence analyses using specific antibodies, as described previously [38].

P815 mouse lymphoblast-like mastocytoma cells (H-2^d^) cultured in the complete DMEM were transfected with pEF1/Neo-NS3 and stable transfectants expressing NS3 were selected using neomycin (G418) (Nacalai Tesque, Kyoto, Japan). The NS3-expressing P815 cells were treated with 25 µg/ml of mitomycin C (Sigma-Aldrich, St. Louis, MO, USA) for 30 min (P815-NS3) and used as stimulator and target cells in a CTL assay using splenocytes obtained from NS3-immunized BALB/c mice (H-2^d^), as described below.

GST-NS3 and GST were produced in *Escherichia coli* BL21 strain and purified with glutathione sepharose 4B beads (GE Healthcare, Buckinghamshire, UK). The proteins were eluted by reduced glutathione in a buffer containing 50 mM Tris-HCl (pH 8.0). After dialysis, the eluted protein was stored at –80°C until being used. The concentrations of purified proteins were determined using Pierce BCA Protein Assay Kit (Thermo Fisher Scientific Inc., Rockford, IL, USA).

### Indirect Immunofluorescence

Cells seeded on glass coverslips in a 24-well plate were fixed with 4% paraformaldehyde in phosphate-buffered saline (PBS) for 15 min at room temperature and permeabilized with 0.1% Triton X-100 in PBS for 15 min at room temperature. After being washed with PBS twice, the cells were consecutively incubated with primary and secondary antibodies. The primary antibodies used were mouse monoclonal antibodies against NS3 (4A-3, a kind gift from Dr. I. Fuke, Research Foundation for Microbial Diseases, Osaka University, Kagawa, Japan) [27]. The secondary antibody used was Alexa Fluor 488-conjugated goat anti-mouse IgG (H+L) (Molecular Probes, Eugene, OR, USA). The stained cells were observed under an All-in-One fluorescence microscope (BZ-9000 Series, Keyence Corporation).

### Immunoblotting

Cells were lysed with SDS sample buffer. Equal amounts of cell lysates were separated by 10% SDS-polyacrylamide gel electrophoresis and transferred onto a polyvinylidene difluoride membrane (Millipore, Bedford, MA, USA), which was then incubated with the respective primary antibodies, followed by incubation with peroxidase-conjugated secondary antibody. The primary antibodies used were mouse monoclonal antibodies against NS3, NS5A and GAPDH (Chemicon International, Temecula, CA, USA). The respective proteins were visualized using ECL immunoblotting detection reagents (GE Healthcare).

### NS3 Serine Protease Assay

Huh-7.5 cells were co-transfected with two plasmids, one expressing NS3 and the other expressing an NS5A/NS5BΔC polyprotein as a substrate, and cultured for 24 h. The cells were lysed and the lysates were subjected to immunoblot analysis using anti-NS5A monoclonal antibody. NS3 serine protease activities were assessed by the cleavage of the NS5A/NS5BΔC polyprotein and emergence of the cleaved-off NS5A [27].

### NS3 Helicase Assay

NS3 helicase activities were determined as described previously with some modifications [39], [40]. In brief, a pair of DNA oligonucleotides (5′-biotin-GCTGACCCTGCTCCCAATCGTAATCTATAGTGTCACCTA-3′ and 5′-digoxygenin-CGATTGGGAGCAGGGTCAGC-3′) were purchased (Operon Biotechnologies K.K., Tokyo, Japan). They were mixed at a 1∶1 molar ratio and annealed to generate a DNA duplex substrate in 50 mM NaCl, 2 mM HEPES, 0.1 mM EDTA and 0.01% SDS by heating at 100°C for 5 min, followed by incubation at 65°C for 30 min and an annealing step at 22°C for 4 h. The DNA duplex substrate (2.5 ng/well) was immobilized via the biotin molecule on the surface of a NeutrAvidin Coated plate (Clear, 8-well strip; Thermo Fisher Scientific Inc.). A reaction mixture (90 µl) containin 11 nM of purified GST-NS3 [26], GST-NS3(K210N) or GST, 25 mM 4-morpholine-propanesulfonic acid (MOPS; pH 7.0), 5 mM ATP, 2 mM DTT, 3 mM MnCl_2_ and 100 µg/ml of bovine serum albumin (BSA) was added to each well. Reactions were carried out for 60 min at 37°C. To stop the reactions, the wells were washed with 150 mM NaCl and dried at room temperature for 15 min. The wells were then washed with a detection washing buffer (100 mM maleic acid, 150 mM NaCl and 0.3% Tween 20, pH 7.5), incubated with a 10% BSA-containing blocking solution (100 mM maleic acid and 150 mM NaCl, pH 7.5) for 30 min followed by incubation with 20 µl of alkaline phosphatase-labeled anti-digoxygenin antibody solution (Roche Applied Science, Germany; 1∶10,000 dilution in the blocking solution) for 30 min. After being washed with a detection buffer (100 mM Tris-HCl, pH 9.5, and 100 mM NaCl), 20 µl of a working solution containing CSPD chemiluminescence substrate (Roche) was added to each well and the plates were incubated for 5 min at 17°C. The wells were then drained and dried, and the luminescence in each well was counted in a luminescence multi-well plate reader. Helicase activities were determined by the reduction of the luminescence, which reflects the release of the digoxygenin-labeled oligonucleotides from the otherwise DNA duplex substrate.

### Luciferase Reporter Assay

Huh-7 cells cultured in a 24-well tissue culture plate were transiently transfected with pSG5-NS3wt or each NS3 mutant (0.25 µg), together with pSG5-NS4A (0.25 µg), pIFN-β-Luc (0.2 µg), pEF1A/N-RIG-I-FLAG (0.05 µg) and pRL-TK (0.01 µg). After 48 h, cells were harvested and a luciferase assay was performed by using Dual-Luciferase Reporter Assay system (Promega). Firefly and Renilla luciferase activities were measured by using a GloMax 96 Microplate Luminometer (Promega).

### Mice and Immunizations

BALB/c mice (H-2^d^) were purchased from CLEA Japan, Inc. Mice were maintained in specific pathogen-free conditions according to institutional guidelines. All of the animal experiments were carried out according to the protocol approved by the Ethics Committee for Animal Experiments at Kobe University (Permit Number: P121002). All surgery was performed under isoflurane anesthesia, and efforts were made to minimize suffering. Eight-week-old female BALB/c mice were immunized with 200 µg of a plasmid, 100 µg each into both quadriceps, by intramuscular injection using a needle-free injector (Twin-Jector EZ II, JCR Pharmaceuticals Co., Ltd., Japan). We adopted the injection dosage according to previous studies [41], [42]. The needle-free jet injection has been reported to enhance the immunological responses induced by DNA vaccines [43]. Mice were boosted with the same plasmid (100 µg) at 4 and 6 weeks after the first injection. Control mice were injected with the empty pSG5 vector.

### Splenocytes Culture

Eight weeks after the first immunization, spleens were resected and crushed with the use of a 22G needle. Splenocytes were strained with a cell strainer (40 µM, BD Falcon, USA) and treated for 5 min with 0.75% ammonium chloride buffer (pH 7.65) to lyse red blood cells. The splenocytes were suspended in RPMI1640 medium supplemented with 2 mM L-glutamine, 10% heat inactivated FCS, 50 U/ml penicillin, 50 U/ml streptomycin and 55 mM 2-mercaptoethanol.

### IFN-γ Secretion Assay

Splenocytes seeded in 96-well (flat-bottom) plates at a concentration of 4×10^5^ cells per well in 200 µl complete medium were stimulated with GST-NS3, or GST as a control, at a concentration of 5 µg/ml for 72 h. The amounts of IFN-γ in the culture supernatants were measured using an ELISA kit (Quantikine Mouse IFN-γ, R&D System, Minneapolis, MN, USA) according to the manufacturer’s instructions.

### Real-time Quantitative RT-PCR

Total RNA was extracted from GST-NS3-stimulated mouse splenocytes using a ReliaPrep RNA cell miniprep system (Promega) according to the manufacturer’s instructions. One µg of total RNA was reverse transcribed using a GoScript Reverse Transcription system (Promega) with random primers and was subjected to quantitative real-time PCR analysis using SYBR Premix Ex Taq (TaKaRa Bio Inc., Kyoto, Japan) in a MicroAmp 96-well reaction plate and an Applied Biosystems 7500 fast Real-time PCR system (Applied Biosystems, Foster City, CA, USA). The primers used to amplify IFN-γ mRNA were 5-CCTGCGGCCTAGCTCTGA-3′ (sense) and 5′-CAGCCAGAAACAGCCATGAG-3′ (antisense). As an internal control, murine glyceraldehyde-3-phosphate dehydrogenase (GAPDH) mRNA levels were measured using primers 5′-CATCGCCTTCCGTGTTCCTA-3′ (sense) and 5′-GCGGCACGTCAGATCCA-3′ (antisense).

### CTL Assay

Splenocytes obtained from NS3-immunized mice were cultured for 5 days with P815-NS3 cells and 5 µg/ml of GST-NS3 to generate effector cells. The effector splenocytes and target P815-NS3 cells (1×10^4^ cells) were cocultured in 96-well plates (round-bottom) for 4 h at 37°C in 5% CO_2_ with ratios of 50∶1, 25∶1, and 12.5∶1. Specific CTL activity was measured using a Lactate Dehydrogenase (LDH) Cytotoxicity Assay Kit (CytoTox 96 Non-Radioactive Cytotoxicity Assay; Promega). Released LDH was measured according to the manufacturer’s protocol. The percentage of specific killing was calculated by the following formula: % specific killing = (experimental release − effector spontaneous release – target spontaneous release)/(target maximum release – target spontaneous release)×100.

### Statistical Analysis

Student’s t-test was used to compare the data between two different groups. For multiple comparisons, a one-way analysis of variance (ANOVA) was used. A *p*-value of <0.05 was considered to be statistically significant.

## Results

### Characterization of Wild Type NS3 (NS3wt) and NS3 Mutants Expressed by DNA Vaccines

**Figure 2:**
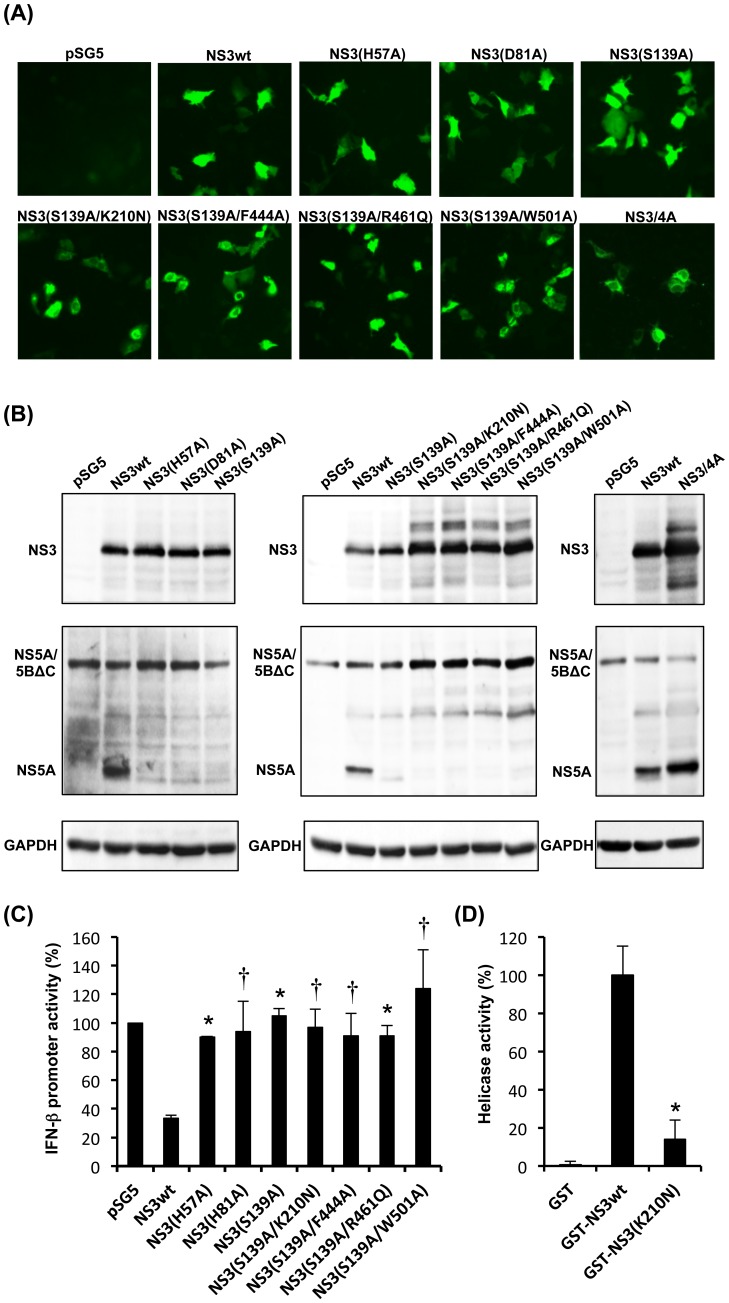
Analysis of NS3 expression, serine protease activity, effects on IFN-β promoter activity and RNA helicase activity. (A) Immunofluorescence analysis of NS3wt, various NS3 mutants and NS3/4A in Huh-7.5 cells transfected with the DNA vaccine candidates using anti-NS3 monoclonal antibody. (B) Serine protease analysis of NS3wt, various NS3 mutants and NS3/4A. Huh-7.5 cells were transiently transfected with each of the NS3 expression plasmids together with pNS5A/5BΔC (as a substrate). Cell lysates were subjected to immunoblot analysis using anti-NS3 and anti-NS5A monoclonal antibodies to detect NS3 (top panel) and NS5A/5BΔC and NS5A (middle panel), respectively. The amounts of GAPDH (bottom panel) were measured as an internal control to verify equal amounts of sample loading. (C) Effects of NS3wt or NS3 mutants on RIG-I-mediated IFN-β promoter activity. Huh-7 cells were transfected with a plasmid expressing NS3wt or each NS3 mutant together with pSG5-NS4A, pEF1A/N-RIG-I-FLAG, pIFN-β-luc and pRL-TK. Firefly luciferase activity was measured 48 h post transfection and normalized to Renilla luciferase activity. Data represent mean ± SEM of the data from three independent experiments. *, *p*<0.01; †, *p*<0.05, compared with NS3wt. (D) RNA helicase analysis of NS3wt and its mutant. NS3 helicase assay was performed using GST-NS3wt, GST-NS3(K210N) and GST as a negative control, as described in the Materials and methods section. The mean activity obtained with the GST control was subtracted from those obtained with test samples. The mean activity of GST-NS3wt was arbitrarily expressed as 100%. *, *p*<0.05, compared with NS3wt.

We constructed plasmids expressing NS3 mutants lacking the serine protease and the NTPase/RNA helicase activities to avoid potential risks posed by those enzymes (Fig. 1). The NS3 mutants were expressed efficiently in Huh-7.5 cells, as demonstrated by immunofluorescence (Fig. 2A) and immunoblotting assays (Fig. 2B, top panel). Importantly, all the NS3 mutants, either protease-deficient single-mutants or protease/helicase-deficient double-mutants, lacked the serine protease activity, as shown by the absence of the cleaved-off product of NS5A (Fig. 2B, middle panel). Equal loading of the samples was verified by GAPDH staining (Fig. 2B, bottom panel). The serine protease activity of NS3 is also known to cleave the RIG-I-associated adaptor protein MAVS (also known as Cardif, IPS-1 and VISA) and, therefore, blockade the RIG-I-mediated induction of IFN-β gene expression [44], [45]. We confirmed that all the NS3 mutants lost their abilities to blockade the RIG-I-mediated IFN-β gene expression (Fig. 2C).

As for the NTPase/RNA helicase activities of NS3, it has been well documented that introduction of either one of the K210N, F444A, R461Q and W501A mutations severely affects the NS3 helicase activity [34], [35], [36]. Indeed, we confirmed that NS3 helicase activity was markedly impaired by the introduction of the K210N mutation (Fig. 2D).

### Induction of IFN-γ Production by NS3-specific T cells after Immunization with NS3 DNA Vaccines

**Figure 3:**
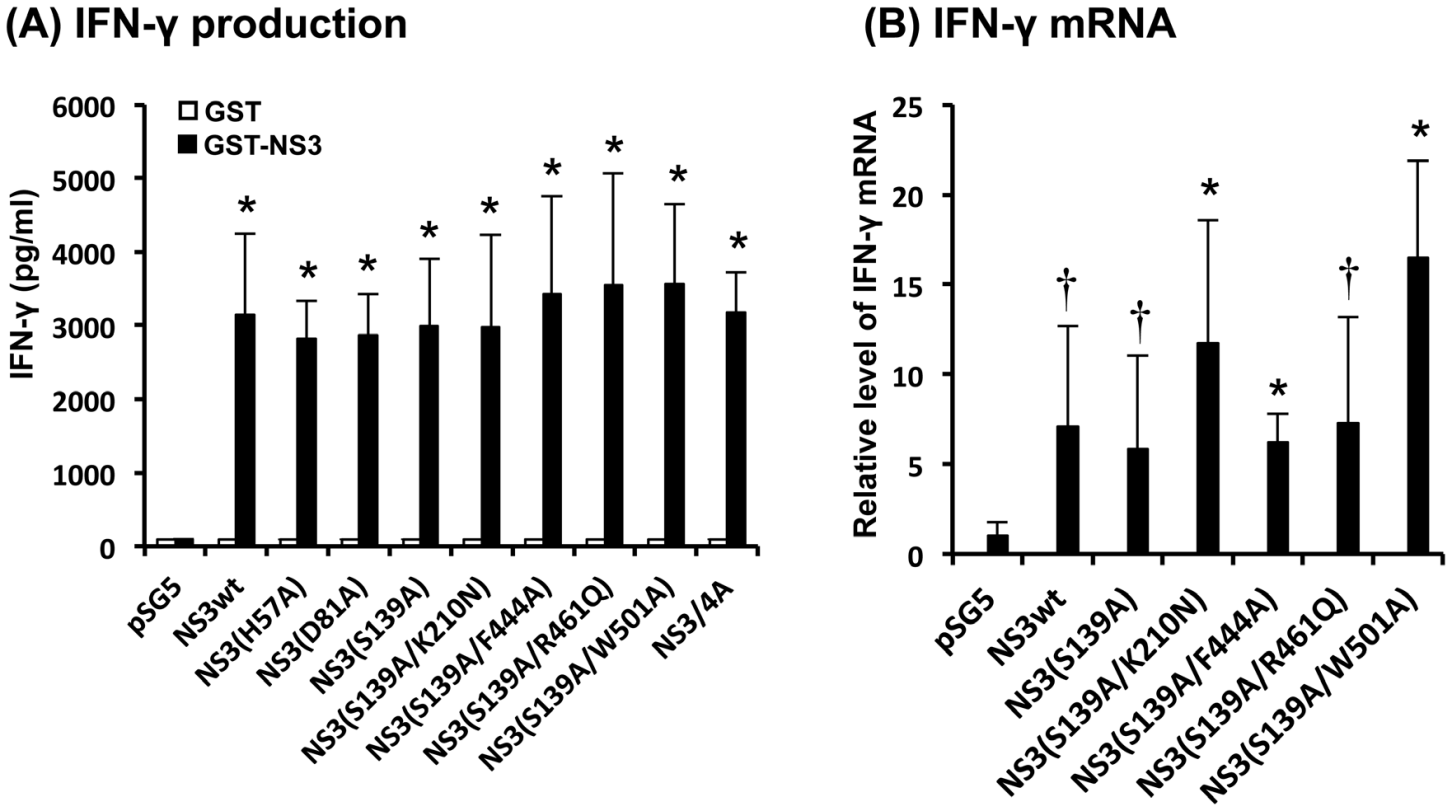
IFN-γ production induced by NS3 DNA vaccination. (A) IFN-γ production by splenocytes obtained from immunized mice. BALB/c mice (2 mice/group) were immunized with each of the DNA vaccines expressing NS3wt, various NS3 mutants or NS3/4A. Splenocytes obtained from the immunized mice were cultured in the presence of GST-NS3 (5 µg/ml) for 72 h. The amounts of IFN-γ in culture supernatants were measured with ELISA. Data represent mean ± SEM of the data from three independent experiments. *, *p*<0.01 compared with the mock-immunized control. (B) IFN-γ mRNA expression. Splenocytes obtained from immunized mice were cultured in the presence of GST-NS3 (5 µg/ml) for 24 h. The amounts of IFN-γ mRNA were determined by real-time quantitative RT-PCR analysis and normalized to GAPDH mRNA expression levels. Data represent mean ± SEM of the data from three independent experiments. The value for splenocytes from the mock-immunized control was arbitrarily expressed as 1.0. *, *p*<0.01; †, *p*<0.05, compared with the control.

In order to evaluate the possible efficacy of the NS3 plasmids as DNA vaccines, BALB/c mice were injected intramuscularly with each of the plasmids, followed by booster injections at 4 and 6 weeks after the first injection. Two weeks after the last immunization, splenocytes were obtained from the mice, stimulated with GST-NS3 *in vitro* and the levels of IFN-γ production in the culture supernatants were measured. The results obtained revealed that protease-deficient single-mutants, i.e., NS3(H57A), NS3(D81A) and NS3(S139A), induced high levels of IFN-γ production, which were comparable to that induced by NS3wt and NS3/4A (Fig. 3A). Moreover, protease/helicase-deficient double-mutants with the backbone of NS3(S139A), i.e., NS3(S139A/K210N), NS3(S139A/F444A), NS3(S139A/R461Q) and NS3(S139A/W501A), induced IFN-γ production to the same extent as observed with the single-mutants. Consistently, real-time quantitative RT-PCR analysis revealed that the levels of IFN-γ mRNA expression were significantly higher in splenocytes obtained from NS3-immunized mice than those from mock-immunized control (Fig. 3B).

### Induction of NS3-specific CTL Activities by Immunization with NS3 DNA Vaccines

**Figure 4:**
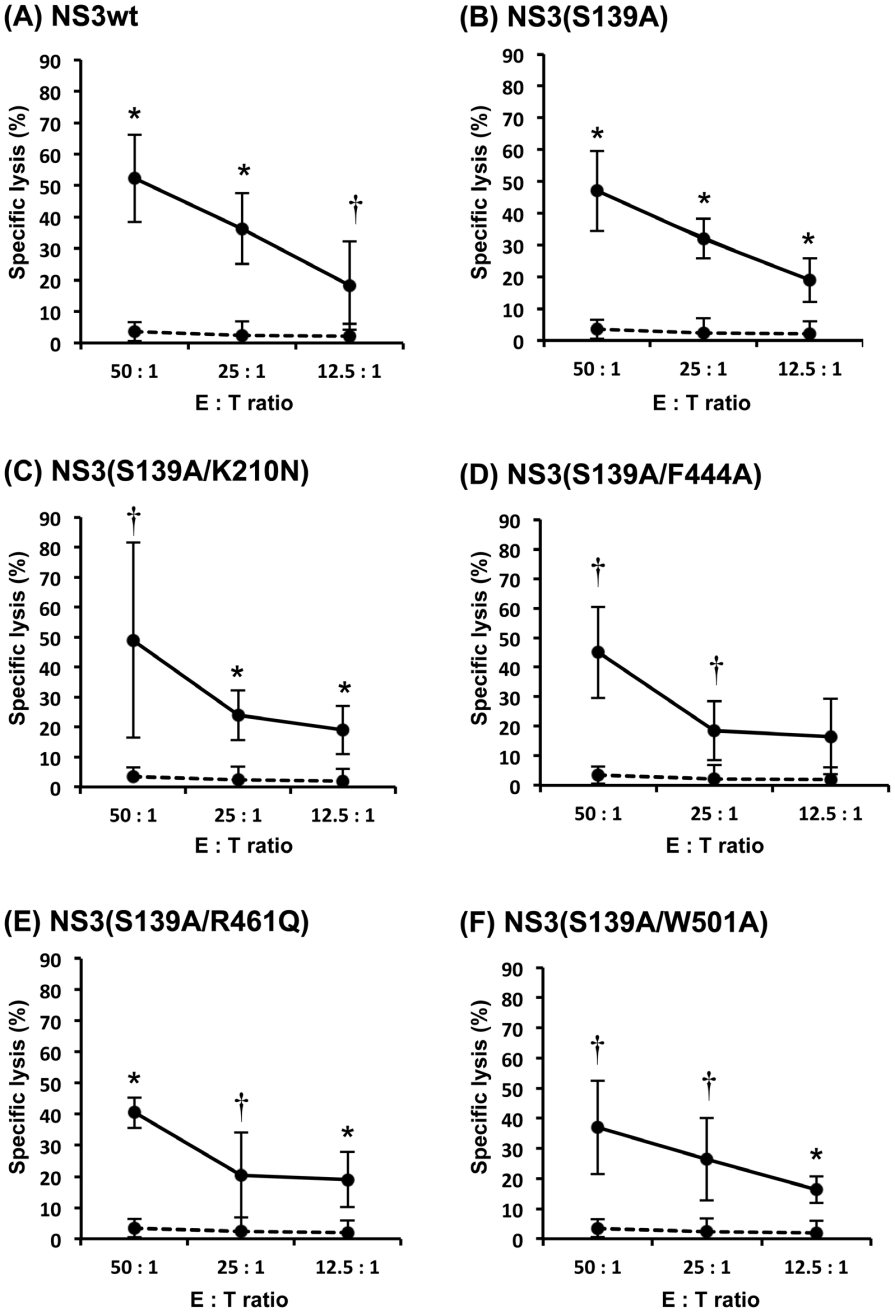
NS3-specific CTL activity induced by DNA vaccination. BALB/c mice (2 mice/group) were immunized with each of the DNA vaccines expressing NS3wt, various NS3 mutants or NS3/4A. Splenocytes obtained from the immunized mice were stimulated in vitro for 5 days with P815-NS3 cells and GST-NS3wt (5 µg/ml). Effectors and targets (P815-NS3) were cocultured for 4 h with the ratios of 50∶1, 25∶1, and 12.5∶1. Released LDH was measured and the percentage of specific killing was calculated. Specific CTL activity of splenocytes obtained from NS3-immunized mice and the mock-immunized control are shown with solid and dashed lines, respectively. Data represent mean ± SEM of the data from three independent experiments. *, *p*<0.01; †, *p*<0.05, compared with the mock-immunized control.

We measured CTL activities induced by the NS3 DNA vaccines. Splenocytes obtained from the vaccinated mice two weeks after the last immunization were stimulated with GST-NS3 and P815-NS3 cells for 5 days and the effector splenocytes were mixed with the target P815-NS3 cells to determine the levels of CTL activities. Protease/helicase-deficient double-mutants, NS3(S139A/K210N), NS3(S139A/F444A), NS3(S139A/R461Q) and NS3(S139A/W501A), induced strong CTL activities against the target P815-NS3 cells to the level equivalent to that induced by NS3wt and a protease-deficient single-mutant NS3(S139A) (Fig. 4).

## Discussion

Effective therapeutic vaccines against virus infection must induce sufficient levels of cell-mediated immune responses against the target viral epitope(s) and also must avoid concomitant risk factors, including potential carcinogenic properties. The HCV NS3 is considered to be an important target for development of HCV therapeutic vaccines because NS3-specific CD4^+^ and CD8^+^ T cell responses correlate well with resolution of the infection [46], [47], [48] and have been described as an indicator for viral clearance both in humans and chimpanzees [48], [49], [50]. On the other hand, NS3 possesses serine protease and NTPase/RNA helicase activities, which are necessary for the viral polyprotein processing and viral RNA replication, respectively [1], [2]. In addition to the essential role in the virus life cycle, the NS3 serine protease interferes with normal cellular functions, such as blockade of IFN-β production [3], [18], [19], [20] and deregulation of EGF signaling [22]. Also, the NTPase/RNA helicase of NS3 may interferes with cellular RNA helicases, which are involved in RNA folding/remodeling [51], enhancement of polymerase processivity [52], and/or genome encapsidation [53]. Importantly, perturbations of cellular RNA helicases are implicated in cancer development [23]. In the present study, therefore, we aimed to develop DNA vaccines that express NS3 mutants lacking both serine protease and NTPase/RNA helicase activities (Fig. 1) in order to avoid concomitant potential risks caused by the viral enzymes.

We first introduced single-point mutations into each of the catalytic triad of the NS3 serine protease (H57A, D81A and S139A) and found that all of the NS3 mutants efficiently induced IFN-γ production by splenocytes obtained from the vaccinated mice (Fig. 3A). Since His at position 57 is located within a well-characterized CD4^+^/CD8^+^ epitope [14], [54], we decided not to choose pNS3(H57A) as a vaccine candidate. We then introduced a point mutation (K210N, F444A, R461Q and W501A) [34], [35], [36] to pNS3(S139A) to impair NTPase/RNA helicase activities. All the resultant DNA vaccine candidates, pNS3(S139A/K210N), pNS3(S139A/F444A), pNS3(S139A/R461Q) and pNS3(S139A/W501A), which express double-mutants lacking both serine protease and NTPase/RNA helicase activities, efficiently induced IFN-γ production by splenocytes of the vaccinated mice. We also observed that the protease-deficient single-mutant pNS3(S139A) and all of the four protease/helicase-deficient double-mutants induced NS3-specific CTL activities to the same extent compared to the non-mutated pNS3wt (Fig. 4). All but H57A mutation of NS3 examined in this study are located outside the human CD4 and CD8 epitopes reported so far, with the H57A mutation being located at the C-terminal edge of an epitope [7], [8], [9], [11]. Therefore, these findings suggest that a single mutation in the protease and NTPase/RNA helicase domains would not interfere with immunogenicity of NS3 as a whole in mice and human.

In general, DNA vaccines mediate antigen expression only transiently in the vaccinees and, therefore, the possible side effects caused by the NS3 enzymatic activities through DNA vaccination would be rather marginal. However, when NS3 is expressed by means of a long-lasting live vaccine, such as a recombinant attenuated varicella zoster virus vaccine, it might potentially exert certain harmful effects after a long period of time. Currently, we aim to generate a recombinant attenuated varicella zoster virus expressing HCV NS3. For this purpose, an NS3 mutant lacking both protease and helicase activities and yet maintaining a full range of antigenic epitopes would be more appropriate than NS3wt.

In summary, we propose that plasmids expressing NS3 protease/helicase-deficient double-mutants, pNS3(S139A/K210N), pNS3(S139A/F444A), pNS3(S139A/R461Q) and pNS3(S139A/W501A), would be good candidates for safe and efficient therapeutic DNA vaccines against HCV infection.
